# Novel *de novo* SPAST mutation in a Han Chinese SPG4 patient: a case report

**DOI:** 10.3389/fgene.2024.1410381

**Published:** 2024-07-30

**Authors:** Yu-Han Xu, Bao-Yu Yuan, Jia-Le Ji, Di Wu, Hong Zhou, Yi-Jing Guo

**Affiliations:** ^1^ School of Medicine, Southeast University, Nanjing, China; ^2^ Department of Neurology, Affiliated Zhongda Hospital of Southeast University, Nanjing, China

**Keywords:** SPG4, hereditary spastic paraplegia, *de novo* variant, late-onset, case report

## Abstract

Spastic paraplegia type 4 (SPG4), the predominant form of Autosomal Dominant Hereditary spastic paraplegia (AD-HSP), is characterized by variants in the SPAST gene. This study reports a unique case of a late-onset SPG4 in a Han Chinese male, manifesting primarily as gait disturbances from lower extremity spasticity. Uncovered through whole-genome sequencing, a previously undocumented frameshift variant, c.1545dupA in exon 14 of the SPAST gene, was identified. Notably, this variant was absent in asymptomatic parents with confirmed paternity and maternity status, suggesting a *de novo* variant occurrence. This discovery emphasizes the potential of *de novo* variants to exhibit a late-onset pure pattern, extending the SPG4 variant spectrum, and consideration of such variants should be given in HSP patients with a negative family history.

## 1 Introduction

Hereditary spastic paraplegia (HSP) encompasses a diverse group of neurodegenerative disorders, marked by genetic and clinical heterogeneity. It has a broad spectrum of causative genes, with approximately 80 susceptible gene loci identified to date. Based on the modes of inheritance, HSP can be divided into autosomal dominant (AD), autosomal recessive (AR), X-linked recessive (XLR), and mitochondrial (Meyyazhagan and Orlacchio, 2022). The hallmark of HSP is the progressive weakness and spasticity of the lower extremities (Shribman et al., 2019). Thus, it can be practically classified into pure and complex forms based on the presence or absence of additional complicating manifestations (Kara et al., 2016). Among these, Spastic paraplegia type 4 (SPG4), caused by variants in the SPAST gene, represents the most prevalent form of autosomal dominant pure HSP (Ebrahimi-Fakhari et al., 2020). This report describes a sporadic Chinese patient with a typical spastic gait and progressive hypermyotonia in the lower extremities. A novel duplication variant (c.1545dup, p.Leu516Thrfs*4) in the SPAST gene was identified, suggesting its *de novo* origin, as it was absent in the patient’s parents. This case highlights the significance of considering *de novo* variants in HSP diagnostics, particularly in patients with no familial disease history.

## 2 Case presentation

### 2.1 The patient

A 35-year-old male noticed unintentional weakness and stiffness in walking 1.5 years ago. On the way up the stairs, he presented an involuntary jerk, which was attributed to the excessive effort. As a result, he did not seek medical advice, and his symptoms gradually worsened, to the point that he felt unsteady even when walking on flat ground. The patient became aware of increasing weakness in both lower extremities over the past 6 months, prompting his visit to our outpatient clinic in July 2022. Prior to symptom onset, he enjoyed good health, and no medical history was noted. Furthermore, he denied that his family had similar symptoms.

Neurological examination revealed a significantly increased muscle tone in the lower extremities with no decreased myodynamia (grade V) and systemic atrophy. In addition, the patient exhibited spastic gait, bilateral hyperreflexia, ankle clonus, and positive Babinski and Hoffmann signs, with preserved intellectual, and no cerebellar, sensory, or autonomic dysfunction was detected.

**FIGURE 1:**
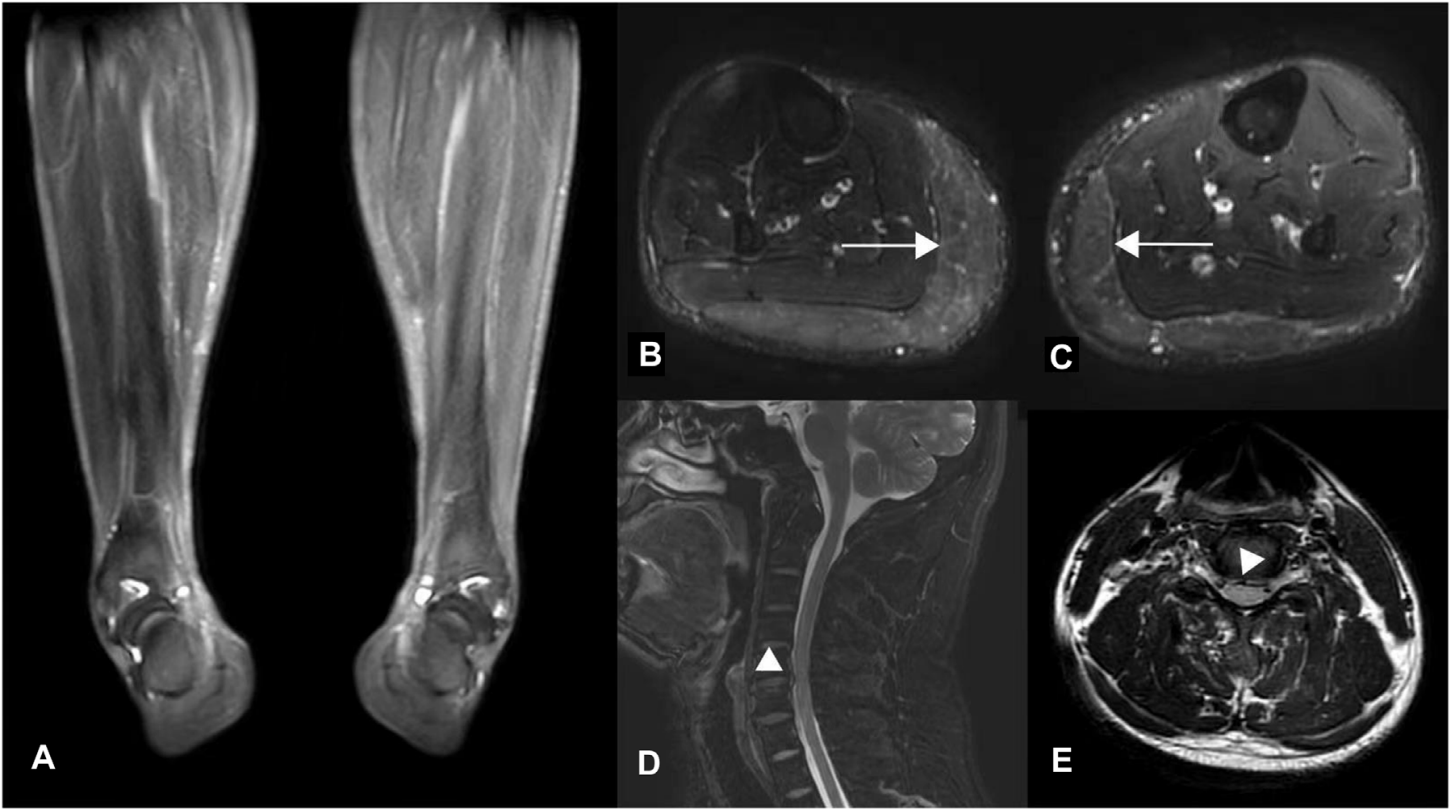
MR images of the proband. **(A)** T2-weighted fat-saturated coronal and **(B, C)** T2-weighted STIR axial images of the lower extremities. Diffuse hyperintensity (horizontal arrows) was seen symmetrically in the bilateral gastrocnemius muscles (Figure B, right lower limb; Figure C, left), which were considered edema due to muscle spasm resulting in the varus deformity of distal limbs, as shown in Figure **(A)**. **(D–E)** Axial and Sagittal T2 flair images of the cervical spinal cord show disc herniation without spinal cord degeneration.

Laboratory analyses, including blood and cerebrospinal fluid tests, showed no abnormalities. Electromyography did not indicate neurogenic lesions, while magnetic resonance imaging (MRI) revealed lateral compartment signal increase in the lower extremities (Figures 1B,C), which were considered edema due to muscle spasms. MRI of the brain was normal; however, the spine MRI showed a herniated cervical disc with compression of the dural sac (Figure 1D). There was no intramedullary degeneration (Figure 1E).

### 2.2 Methods

Blood samples were collected from family members, and genomic DNA was extracted following the manufacturer’s recommended protocol using the QIAamp DNA Blood Midi Kit (Qiagen, Hilden, Germany). DNA concentrations were quantified using the dsDNA BR Assay on a Qubit Fluorometer (Thermo Fisher Scientific, Carlsbad, CA, USA). Genomic DNA was fragmented with Hieff Smearase (Yeasen, Shanghai, China) and selectively enriched for fragments ranging from 200 to 500 bp using DNA clean beads. Library preparations were carried out using the Hieff NGS OnePot DNA Library Prep Kit for Illumina (Yeasen, Shanghai, China). Next-generation sequencing (paired-end 150 bp) was performed using DNBSEQ-T7 (MGI, Shengzhen, China).

Upon receipt of the original sequencing data, we performed data processing and bioinformatics analysis to detect variants. Afterward, we used Fastp (Chen et al., 2018) to generate “clean reads” for further analysis. The “clean reads” (with a length of 150 bp) derived from targeted sequencing and filtering were then aligned to the human genome reference (hg19) using the BWA (Burrows-Wheeler Aligner) software (Li and Durbin, 2009). After alignment, output files were used to perform sequencing coverage and depth analysis.

We used GATK (Genome Analysis Toolkit) software to detect single nucleotide variants (SNVs) and indels. All SNVs and indels were filtered and estimated *via* multiple databases, including 1000 Genomes (1000 human genome dataset), Genome AD (Genome Aggregation Database dataset), and ExAC (The Exome Aggregation Consortium dataset). We used dbNSFP (Liu et al., 2016) to predict the effect of missense variants. The Human Gene Mutation Database (HGMD) and Clinvar Database were used to screen variants reported in published studies. Pathogenic variant assessments followed the guidelines established by the American College of Medical Genetics and Genomics (ACMG) (Richards et al., 2015), with all suspected pathogenic variants being validated through Sanger sequencing. Structural Variants (SVs) were identified using LUMPY (Layer et al., 2014).

Considering the identification of a *de novo* variant in SPAST, a short tandem repeat (STR) analysis was performed in the proband and the unaffected parents to confirm the parenthood using the HUMDNA TYPING (Yanhuang) Kit (FGI, Shengzhen, China). Twenty-six polymorphic autosomal STR markers commonly used for paternity testing in Chinese populations and a sex-identification marker (Amelogenin locus) were detected, including D3S1358, D13S317, D7S820, D16S539, SE33, D10S1248, D5S818, D21S11, TPOX, D1S1656, D6S1043, DXS6795, D19S433, D22S1045, D8S1179, Penta E, DYS391, D2S441, D12S391, D2S1338, vWA, Penta D, TH01, D18S51, CSF1PO, FGA.

All procedures were conducted under informed consent from the patient and his parents.

## 3 Results

### 3.1 Clinical analysis

**FIGURE 2:**
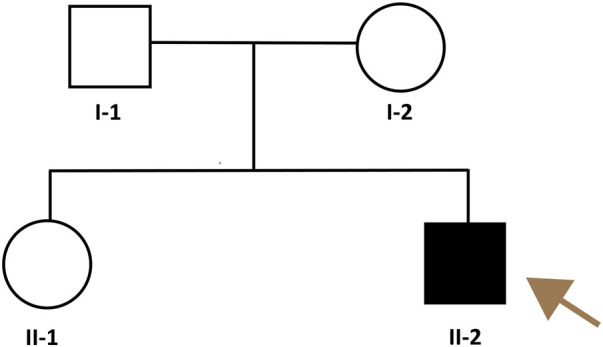
Pedigree of the HSP family. The brownish arrow indicates the proband. Squares indicate men; circles, women; shaded (black) symbol, individual with HSP, unshaded symbols, individuals without HSP.

The pedigree of the family is shown in Figure 2. The patient had symmetrical upper motor neuron syndrome (UMNS) in the lower extremities, and the upper limbs were less affected. Even though cervical spondylosis was present, he had no clinical abnormalities of limb sensation. Thus, the pyramidal sign in this patient cannot be explained solely by cervical spondylosis. At this point of the investigation, we concluded a slowly progressive UMNS exclusively involving lower limbs without any apparent biological or radiological cause suggesting the possibility of HSP and Primary lateral sclerosis (PLS). According to the unspecific clinical and paraclinical examinations, we carried out genetic tests on the family despite the negative family history.

### 3.2 Molecular genetic findings

**FIGURE 3:**
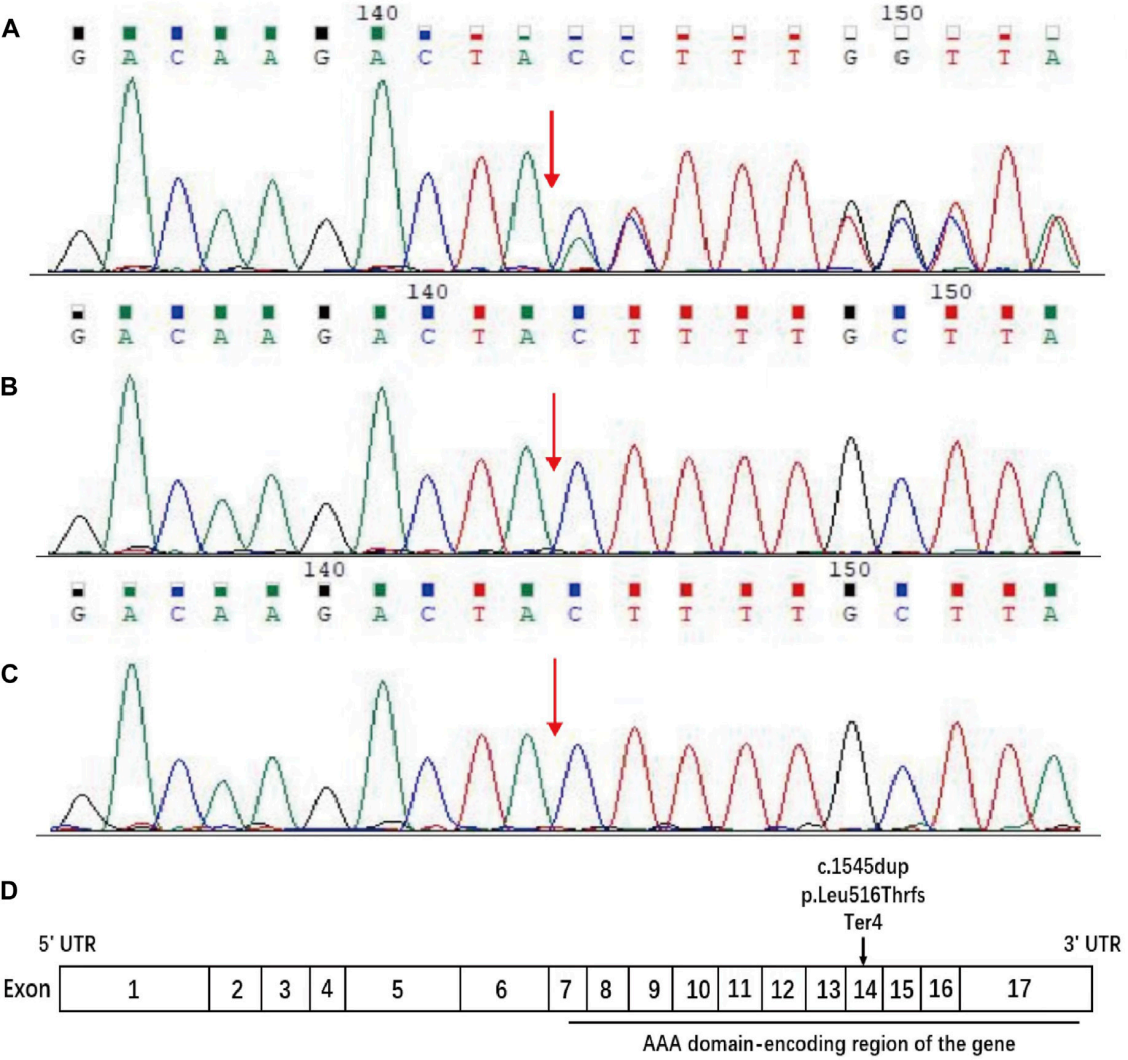
Sequence analysis of the SPAST gene. DNA samples were provided for sequencing by the patient and his parents. **(A)** The patient carried c.1545dupA in exon 14 of SPAST, resulting in the change of (p. Leu516Thr) leucine (CTT) to threonine (ACT) and a frameshift variant that led to early stop codon emergence. **(B, C)** The patient’s father and mother have no variant at this site. The red arrow indicates the position of the C 1545 nucleotide. **(D)** Schematic diagram of the structure of spastin. The horizontal black line indicates the AAA domain-encoding region of the gene, and the arrow indicates the position of the current variant.

**FIGURE 4:**
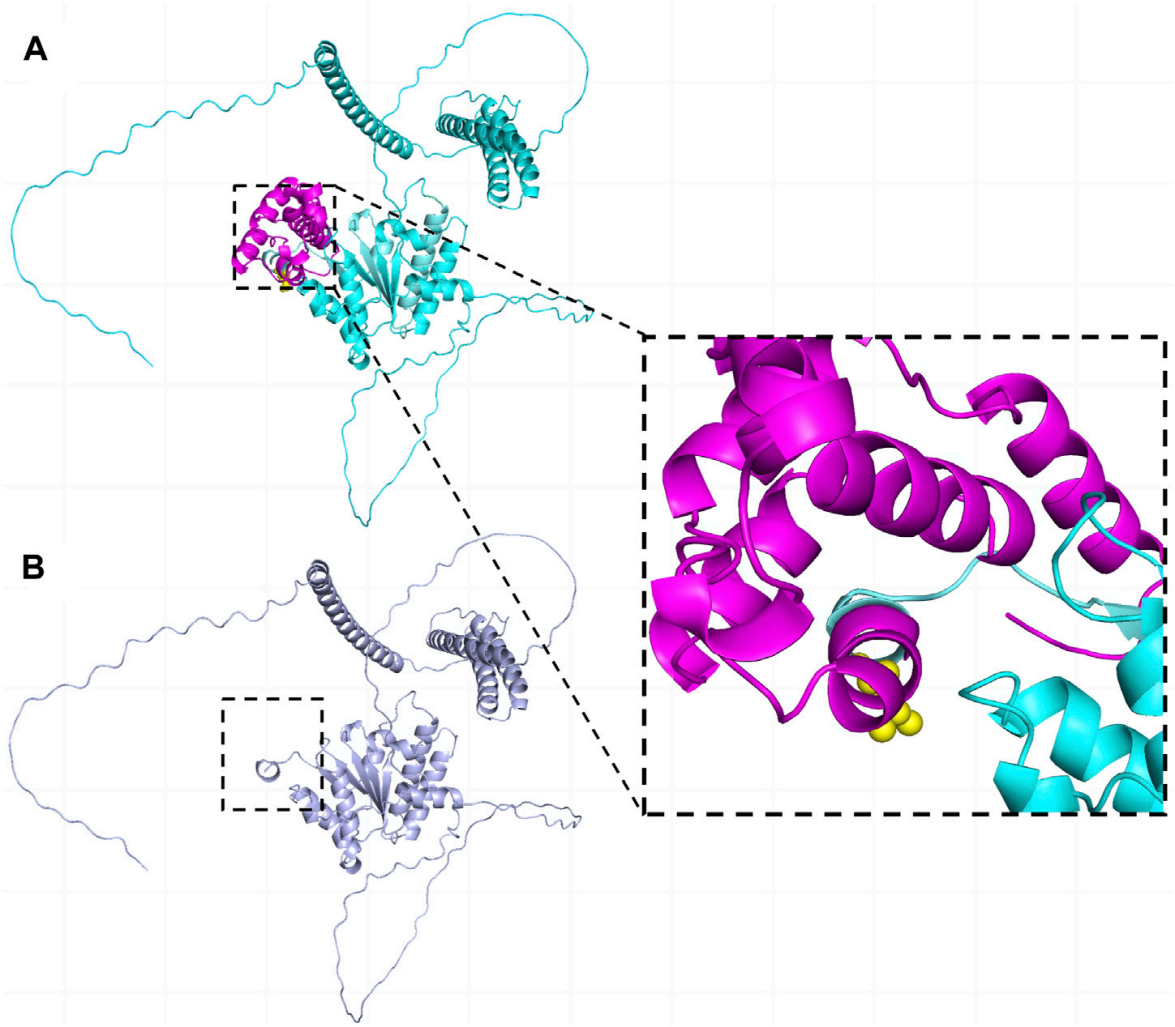
The effect of the variant (p.Leu516Thrfs*4) on SPAST protein structure. The structures were modeled on the basis of Swiss-model (https://swissmodel.expasy.org/) and PyMOL3.0. **(A)** Wild-type. Leu516 was shown as yellow spheres in the zoomed-in view. **(B)** The truncated protein. The structure marked in rose red was deleted.

Sequences analysis revealed a heterozygous variant in exon 14 of SPAST (c.1545dupA), causing a frameshift variant (PVS1) with an early stop codon (p.Leu516Thrfs*4). The variant was reconfirmed using Sanger sequencing (Figure 3A), and its structural impact was further analyzed through Swiss-model (Figure 4). It was not found in the databases1000 Genomes (http://www.international-genome.org) or gnomAD (http://gnomad.broadinstitute.org) (PM2). Family co-segregation analysis showed that the variant was only detected in the proband with confirmed family relation (Figures 3B,C) (PS2). Collectively, we identified the ACMG criteria for this variant as PVS1, PM2, and PS2, indicating pathogenic.

On the other hand, we perform a paternity test. The numbers of repeats in each STR marker in the family members are shown in Table 1. Although there might be some microsatellite instability, the bio-statistical computations strongly supported the maternity relationship. As a result, both paternity and maternity of the parents were confirmed in this case; thus, the variant occurred *de novo* in the patient.

**TABLE 1:** Paternity and Maternity Testing by analysis of Forensic Short Tandem Repeat (STR) Markers in the Family Members.

	Son		Father		Mother	
D3S1358	15	16	16	17	15	null
D13S317	8	11	8	9	11	null
D7S820	8	12	12	null	8	11
D16S539	12	null	12	null	10	12
SE33	20	29.2	20	27.2	24.2	29.2
D10S1248	13	14	14	17	13	15
D5S818	11	12	11	12	11	12
D21S11	30	32.2	32.2	33.2	30	31.2
TPOX	8	11	8	11	8	9
D1S1656	13	null	13	15	11	13
D6S1043	13	17	13	17	11	13
DXS6795	13	null	10	null	13	null
D19S433	13	14.2	13	14	13	14.2
D22S1045	11	15	15	16	11	15
D8S1179	13	14	12	14	13	15
Penta E	10	15	11	15	10	18
DYS391	10	null	10	null	null	null
D2S441	11	null	11	null	11	14
D12S391	19	23	19	20	18	23
D2S1338	19	23	18	19	23	24
vWA	16	null	16	19	14	16
Penta D	6	13	6	9	12	13
TH01	6	7	6	null	7	9
D18S51	14	24	14	null	19	24
CSF1PO	9	13	9	10	12	13
FGA	22	23	23	24	22	null
Amelo	X	Y	X	Y	X	X

## 4 Discussion

Hereditary spastic paraplegia (HSP), also known as Strümpell–Lorrain disease, is characterized by progressive muscle weakness and spasticity in both lower extremities. Due to its complex clinical phenotype and pronounced genetic heterogeneity, it brings significant challenges to clinical practice, and the diagnosis requires confirmation by DNA analysis. Although neurological testing shows pyramidal syndrome, such as hypermyotonia and hyperreflexia, care must be taken to distinguish these from other causes of spasmodic paraplegia. In the present study, we encountered a late-onset pure SPG4 phenotype caused by a *de novo* mutation in the SPAST gene in a Chinese patient. Direct sequencing of the SPAST gene revealed the novel variant in a heterozygous state in the patient with a pure form of HSP.

SPAST (located on 2p22.3) is the most frequently mutated gene of HSP, accounting for 15%–40% of all HSP cases. It spans the 90 kb region of genomic DNA containing 17 exons (Figure 3D) and encodes an ATPase called spastin (Hazan et al., 1999). Spastin is a member of the AAA (ATPase associated with various cellular activities) protein family that breaks longer microtubules into shorter and more dynamic ones, thereby regulating the number and mobility of microtubules and the distribution of their dynamic plus-ends (Errico et al., 2002). More than 200 variants have been identified within the AAA domain in SPG4 patients (Cui et al., 2020). Naturally, inadequate microtubule severing resulting from haploinsufficiency has been proposed as the mechanism underlying SPG4 (McDermott et al., 2003; Blackstone, 2018; Behne et al., 2020). However, there is growing evidence that insufficient microtubule severing alone is not an adequate explanation for SPG4 (Solowska and Baas, 2015; Parodi et al., 2018). Chen et al. (2022) found that truncated spastin may damage the corticospinal tracts through an isoform-specific toxic effect.

In this case, the patient had a variant caused by c.1545dupA, which has not yet been reported in the literature. The variant introduced a premature termination codon, suggesting a dual impact on protein function that may entail both loss-of-function and potential gain-of-function aspects. Ultimately, it resulted in haploinsufficiency and gave rise to truncated protein isoforms, which were considered likely pathogenic.

The type of genetic variant in SPG4 patients was strongly associated with age and disease severity. For example, clinical symptoms of most missense variant carriers first appeared before the second decade, whereas the first clinical symptom onset of truncating variant carriers clustered between the second and sixth decades. Meanwhile, the disability and progression for late-onset cases were more severe and rapid than those of early-onsets (Parodi et al., 2018). Chelban et al. (2017) found that truncating variants in SPAST patients are associated with a high rate of psychiatric comorbidities. Our patient fitted the late-onset profile, as he did not start getting sick until he was in his 30s. However, he has not shown any psychiatric symptoms yet. Rehabilitation therapy was initiated after diagnosis, even though at the last follow-up, symptoms such as gait stiffness had worsened. There seems to be an acceleration in disease progression. Hence, the relationship between SPAST variant types and clinical phenotypes should be a matter of concern.

This variant was a *de novo* event, as both parents exhibited normal sequencing. Schieving et al. (2019) pointed that the majority of patients (22/27, 81%) with *de novo* variants have an extremely early onset (<10 years) of the disease and that the complex types exhibited more severe neurological symptoms than in familial cases. Another study reconfirmed that *de novo* variants in SPAST usually lead to a severe and complex form of HSP (Mo et al., 2022). However, this finding is inapplicable to our patient, and the association between genotype (truncating variant arisen *de novo*) and phenotype (age of onset and the form of HSP) may require further investigation.

In conclusion, anyone with progressive UMNS in lower limbs should consider HSP after ruling out obvious causes. Early diagnosis will lead to early rehabilitation therapies to improve muscle tone and maintain flexibility. The final diagnosis was based on the identification of the mutated genome. Here, we report a novel heterozygous *de novo* variant in the SPAST gene that results in the frameshift variant and truncation of the protein. This study could help expanding the knowledge about the clinical parameters and variant spectrum of SPG4.

## Data Availability

The datasets presented in this article are not readily available because of ethical and privacy restrictions. Requests to access the datasets should be directed to the corresponding author.
